# Tailoring, spectroscopic, DFT, solvatochromic, antitumor, and molecular docking studies of new polynuclear Cu(II)-hydrazone complexes

**DOI:** 10.1038/s41598-025-32666-8

**Published:** 2026-01-06

**Authors:** Fatma Samy, Magdy Shebl, Ebtesam M. Abdelrhman

**Affiliations:** Department of Chemistry, Faculty of Education, Ain Shams University, Roxy, Cairo, Egypt

**Keywords:** Copper(II) complexes, Hydrazones, 4,6-Diacetylresorcinol, DFT calculations, Anticancer activity, Molecular docking, Cancer, Chemistry

## Abstract

Hydrazones and their metal complexes have acquired a lot of interest because of their various biological and catalytic applications. The reaction of the symmetrical hydrazone (NBHD) with Cu(II) salts, including Cl^−^, Br^−^, and SO_4_^2−^, has been investigated. The structures of Cu-NBHD complexes were explored by using various spectroscopic and analytical tools. Fluorescence spectra for NBHD and Cu(II)-NBHD complexes were recorded in a large number of solvents to probe their solvatochromic manners. Theoretical calculations for NBHD and Cu-NBHD complexes were conducted, and the results were correlated with the experimental data. The anticancer action of Cu-NBHD complexes was investigated towards Hepatocellular carcinoma and the results were supported by molecular docking studies. The Cu-NBHD complexes have distorted octahedral geometrical structures as evidenced from magnetic moment, electronic and ESR spectral data. NBHD acts as a *bis*(monoanionic bidentate) in case of Cl^−^ and Br^−^ ions and *bis*(neutral bidentate) in case of SO_4_^2−^ ion. The coordinating sites are phenolic oxygen and azomethine nitrogen atoms. In case of bromo and sulfato complexes, binuclear complexes were obtained. However, a tetranuclear complex was obtained in case of the chloro complex. DFT calculations for NBHD and Cu-NBHD complexes were performed, and the results were correlated with the practical results. All Cu-NBHD complexes exhibited anticancer activity towards Hepatocellular carcinoma. The bromo complex **2** showed an enhanced activity than that of *cis*Pt. Using different copper(II) salts gives different bi- and tetra-nuclear complexes. Al complexes exhibited anticancer activity towards Hepatocellular carcinoma and the bromo complex **2** showed enhanced activity than that of *cis*Pt. The encouraging activity prompts further studies about the complex as an antitumor agent.

**Supplementary Information:**

The online version contains supplementary material available at 10.1038/s41598-025-32666-8.

## Introduction

Design and manufacture of hydrazones have gained significant and continuing curiosity for a long time. Hydrazones have many applications, such as anticancer^1^, DNA cleavage^2^, antioxidant^2^, antimalarial^3^, antimicrobial^3^, antiviral^4^, and antihypertensive^5^ agents. The hydrazonoic complexes show a number of useful properties, including the ability to catalyze chemical reactions^6^, fluorescent^7–10^, optoelectronic^11^, analytical^12–17^, and biological and pharmaceutical^18–32^ properties.

Transition elements have a significant function in therapeutic biochemistry. There has been a lot of interest in the layout and preparation of transition metal chelates^33–39^. These complexes are now widely used for various biological and pharmacological purposes^33–36^. In addition, they are frequently employed in spectrophotometry, chromatography, and electrophoresis^40,41^.

It’s interesting to note that complexes containing hydrazone ligands and metal ions, including copper ions, frequently have greater antitumor activity than the hydrazone ligands themselves^27,28,31^. Consequently, the formation of copper complexes with hydrazones is very important. In addition, copper complexes have anticancer properties by regulating the tumor microenvironment, preventing tumor angiogenesis and metastasis^42^, and raising the immune system’s capacity to destroy cancer cells as well as the powerful DNA-binding capacity of Cu(II) ions^43,44^. Polynuclear complexes have garnered a lot of interest over the years^45,46^. The numerous applications pertaining to the various divisions of the natural sciences—chemistry, biology, and physics—are the source of this continuous interest^47^.

The coordinating manner of 4,6-*bis*(1-hydrazonoethyl)benzene-1,3-diol (BISHD) with metal ions has been investigated^48^. Furthermore, symmetrical chelating agents were synthesized using BISHD^27,28,49^. A *bis*(bidentate) ligand known as 4,6-bis(3-nitrobenzalen-1-yl)methylene)hydrazono)-ethyl)benzene-1,3-diol (NBHD) was synthesized, and its coordinating manner toward Cu(II), Ni(II), and Co(II) acetates was examined^27^. The ligand and its complexes showed antitumor activity against Hepatocellular carcinoma. Complexation process improved the antitumor activity, and copper (II) complex showed a promising activity. A *bis*(tridentate) ligand was synthesized by the reaction of BISHD with 2-hydroxy 1-naphthaldehyde^28^. Reactions of the ligand with Co(II), Ni(II), and Cu(II) ions have been carried out giving a series of binuclear complexes. The prepared compounds showed anticancer activity toward Hepatocellular carcinoma cells. Interestingly, Shebl^49^ has synthesized three polydentate ligands by reaction of BISHD with furan-2-carboxaldehyde, thiophene-2-carboxaldehyde and 1 H pyrrole-2-carboxaldehyde, respectively. The coordination behavior of the ligands toward cobalt(II), nickel(II), copper(II), zinc(II), cadmium(II), and dioxouranium(VI) ions was explored. Different geometrical arrangements were proposed for metal complexes. The antitumor activity of the synthesized compounds was tested on Ehrlich Ascites Carcinoma. Copper(II) complexes showed promising IC_50_ values, which are analogous to that of *cis*platin. Additionally, Elsayed et al.^50^ have synthesized a symmetrical multidentate ligand by reaction of BISHD with isatin. Reaction of the ligand with copper(II), cobalt(II), nickel(II), and zinc(II) ions yielded mono- and bi-nuclear complexes with different geometrical structures. The ligand and some metal complexes showed antimicrobial activity. It was clearly noted that all complexes based on BISHD exhibited promising biological activities, particularly the copper(II) complexes. However, the influence of anions on complex formation and their subsequent antitumor activity remains unexamined.

The goal of the current investigation is to determine how the counter anions affect the ligational behavior of the hydrazone (NBHD) as well as the kind and shape of the isolated copper(II)-complexes. Furthermore, the antitumor activity of Cu(II)-NBHD complexes was explored and correlated with molecular docking study.

## Methodology

### Reagents and materials

The following were BDH or Merck products: copper(II) salts, hydrazine hydrate, 3-nitrobenzaldehyde, acetic anhydride, resorcinol, zinc(II) chloride, metal indicators, zinc(II) sulphate, and conc. HNO_3_. Chemicals of reagent grade, organic solvents were used as originally provided.

### Synthesis of Cu(II)-NBHD complexes

4,6-Diacetylresorcinol^51^, 4,6-bis(1-hydrazonoethyl)benzene-1,3-diol (BISHD)^49^, and 4,6-bis(3-nitrobenzalen-1-yl)methylene)hydrazono)-ethyl)-benzene-1,3-diol (NBHD)^27^ were prepared following literature methods.

#### Complex 1

A solution of CuCl_2_.2H_2_O (0.28 g; 1.64 mmol in ~ 50 mL ethanol) was poured slowly to NBHD (0.4 g; 0.82 mmol in ~ 50 mL ethanol) and the mixture was heated to reflux for ~ 11 h. After cooling, the pale green precipitate was filtered off, washed with ethanol then diethyl ether and finally kept in a desiccator (yield = 0.71 g; 74%).

#### Complex 2

A solution of CuBr_2_ (0.367 g; 1.64 mmol in ~ 50 mL ethanol) was poured slowly to NBHD (0.4 g; 0.82 mmol in ~ 50 mL ethanol) and the mixture was heated to reflux for ~ 11 h. After cooling, the yellowish green precipitate was filtered off, washed with ethanol then diethyl ether and finally kept in a desiccator (yield = 0.61 g; 71%).

#### Complex 3

A solution of CuSO_4_.5H_2_O (0.41 g; 1.64 mmol in ~ 60 mL ethanol-water mixture) was poured slowly to NBHD (0.4 g; 0.82 mmol in ~ 50 mL ethanol) and the mixture was heated to reflux for ~ 11 h. After cooling, the brownish orange precipitate was filtered off, washed with ethanol-water mixture then diethyl ether and finally kept in a desiccator (yield = 0.5 g; 70%).

### Instrumentation

Supplementary materials collected the instrumentation utilized in the current work.

### DFT calculations

All theoretical calculations were conducted using ORCA 6.1.0. NBHD and Cu(II)-NBHD complexes were optimized at B3LYP^52–54^ with 6-311G(d, p)^55^ basis set for C, H, N, O, S, Cl, and Br atoms and a LANL2DZ^56,57^ for copper atom. The reported equations in literature^58^ were employed to calculate essential chemical parameters.

### Biological and Docking studies

The anticancer activity of Cu-NBHD complexes was tested against Hepatocellular carcinoma cells (HepG-2) as well as normal Human embryonic kidney cells (HEK-2) at Al-Azhar University’s regional center for (mycology & biotechnology), using the standard literature technique^59^. The Hepatocellular carcinoma cells were purchased from VACSERA Tissue Culture Unit. The method used for antitumor assay is provided in detail in supplementary materials.

The studied compounds were subjected to molecular docking simulations using Autodock and Autodock tools^60^. The dimensional of the search box was (X = 30, Y = 11, Z = 33 Å), and size dimensional of (X = 18, Y = 23, Z = 13 Å). Molecular docking was utilized to investigate the binding processes of NBHD and Cu(II)-NBHD complexes against the crystal structure of the CDK-5 inhibitor EFP bound to the CDK-2 receptor (PDB ID: 3IG7). The three-dimensional structure of the target receptor was got by downloading it from the Protein Data Bank (http://www.rcsb.org/). The investigated compounds were ranked based on their affinity for binding to the target receptor using the binding energy (kcal/mol).

## Results and discussion

The characteristic physical and analytical data of Cu-NBHD complexes are collected in Table 1. The Cu-NBHD complexes are green and brownish orange in color and non-hygroscopic. They are sparingly soluble in water and most common organic solvents except DMF or DMSO.

The Cu-NBHD complexes have neutral (non-electrolytes) properties^61^ due to the molar conductance values in the range 2.1–11.7 Ω^−1^ cm^2^ mol^−1^. The deprotonation of the phenolic-OH groups upon coordination with copper(II) ion and/or the direct connection of the anions (chloride, bromide or sulfate) to the copper(II) ion in the inner coordination sphere is what gives the Cu-NBHD complexes their neutral characteristics. The FT infra-red data confirms this finding.

**Table 1:** Important physicochemical properties and analytical data of the Cu-NBHD complexes (**1**–**3**).

	Reactants	Complex M.F. [F. Wt]	Color	%Yield	M.*P*. °C	Elemental analysis, % Found/(Calc.) C	H	*N*	M
**1**	NBHD (H_2_L) + CuCl_2_	[Cu_4_(L)Cl_6_(H_2_O)_10_].2H_2_O [1169.5] C_24_H_42_N_6_O_18_Cl_6_Cu_4_	Pale green	74	> 300	24.28 (24.65)	3.52 (3.62)	7.23 (7.19)	21.41 (21.73)
**2**	NBHD (H_2_L) + CuBr_2_	[Cu_2_(L)Br_2_(EtOH)_6_] [1049.74] C_24_H_54_N_6_O_12_Br_2_Cu_2_	Yellowish green	71	243^a^	41.38 (41.19)	4.95 (5.19)	8.19 (8.01)	12.02 (12.11)
**3**	NBHD (H_2_L) + CuSO_4_	[Cu_2_(H_2_L)(SO_4_)_2_(H_2_O)_4_] [879.72] C_24_H_28_N_6_O_18_S_2_Cu_2_	Brownish orange	70	248^a^	32.54 (32.77)	3.20 (3.21)	9.38 (9.55)	14.12 (14.45)

^a^ Shrinking.

### FT-IR spectra

The fundamental FT-IR spectral data of the Cu-NBHD complexes are recorded in Table 2. Supplementary materials (Fig. S1 and S2) depict the ^1^H NMR and FT-IR spectra of NBHD and its complexes. The FT-IR spectrum of NBHD is compared with Cu-NBHD complexes giving the next remarks; (i) Broad absorption bands within the range 3346–3485 cm^−1^ were recognized in Cu-NBHD complexes, which may be due to ν(OH) of the coordinated or solvated/non-coordinated ethanol and/or water molecules attached to Cu(II) ions in the Cu-NBHD complexes. (ii) The band assigned to ν(C = N)azomethine exhibited a relatively slight shift to lower wavenumber (5–12 cm^−1^) whereas the band assigned to ν(C-O)phenolic exhibited a pronounce shift (17–31 cm^−1^) in the Cu-NBHD complexes, revealing contribution of these coordinating sites in coordination^62^. (iii) The noticeable shift in the band assigned to ν(C-O)_phenolic_ following complexation with copper(II) ion is correlated with the characteristic Lewis basicity of the coordinated anion^63^. The red shift of νC-O/cm^−1^ band of the present copper(II) complexes boosted by the donating ability of anion (A) as indicated from the positive slope of the linear correlation of the stretching frequency of C-O *versus* the corresponding ΔE gap (analogous to stability) from DFT calculation (*vide infra*), νC-O/cm^−1^ = 981.93 + 26.781ΔE/eV, R^2^ = 0.96, *n* = 4. Furthermore, the positive slope of C = N/Å= 1.507 + 0.0565 E_Lumo_/eV, R^2^ = 0.91, *n* = 3; extent of C-N elongation as a result of azomethine coordination correlated with the complex stability (higher E_Lumo_). The formation of M-N and M-O coordinate bonds are further confirmed by the existence of new bands in the 427–443 and 515–559 cm^−1^ ranges, which may be assigned to ν(Cu-N) and ν(Cu-O) bands, correspondingly^64–69^. The data reveals the shortness of Cu–O and Cu-N bond lengths, were accompanied by the elongation of (C-O)phenolate and (C = N) azomethine bond lengths. In other words, the Cu–O and Cu-N bond strengths are found to be inversely proportional with the Lewis basicity of anion (Cu–A); A = Cl^–^ and Br^–^ have donor numbers (DN_A_) 36.2 and 33.7, respectively^63^. This is indicated by the range of red shift of ν(C-O) to 17 and 31 cm^−1^ for chloro- complex **1** and bromo complex **2**), which is in an excellent accord with Gutmann bond’s variation rules that stated “elongation followed by shortness”^70^. (iv) The existence of new bands for complex (**3**) at 1104 and 622 cm^−1^ can be assigned to the bidentate SO_4_^2-^ group^71^. In summary, the change in position and intensity of the C-O and C = N bands, along with the appearance of new M-O and M-N bands, are the primary IR indicators that coordination of phenolate and azomethine groups making dynamic redistribution of charges in a metal complexes.

**Table 2:** Specific FT infra-red spectroscopic bands of NBHD and Cu-NBHD complexes.

No.	IR Spectra (cm^− 1^)								
	ν(OH) phenolic/H_2_O/EtOH	ν(C-H) aromatic	ν(C-H) aliphatic	ν(C = N) azomethine	ν(NO_2_)sym	ν(C-O) phenolic	ν(M-O)	ν(M-N)	Other bands
NBHD [27]	3139	3077	2971	1597	1350	1076	-	-	-
**1**	3346	3076	2926	1585	1351	1059	515	443	-
**2**	3418	3078	2924	1592	1352	1045	559	427	-
**3**	3485	3075	2924	1592	1351	1045	522	429	1104, 622

### Magnetic moment and electronic spectra measurements

Table 3 records the electronic spectroscopic results of Cu-NBHD complexes and Fig. S3 (Supplementary materials) depicts the spectra of NBHD and its complexes. The absorption bands in the range 647–691 nm are noticed in the electronic spectra of the Cu-NBHD complexes (**1–3**), which may be ascribed to the ^2^E_g_ → ^2^T_2g_ transition in a distorted octahedral geometrical arrangement.

The calculated magnetic moments of Cu-NBHD complexes (see Table 3) are within the range 1.32–1.66 BM, demonstrating the presence of an unpaired electron^7,72^. The lower values than the recognized for Cu(II) complexes may be due to the strong interaction of Cu(II) with adjacent central ions^72^. Thus, electronic spectra and magnetic moment measurements revealed octahedral geometry.

**Table 3:** Magnetic moments, electronic spectra, and molar conductance data of Cu(II)-NBHD complexes (**1**–**3**).

No.	µ_complex_ B.M.	µ_eff_ B.M.	Conductance ^a^ (Ω^−1^ cm^2^ mol^− 1^)	Electronic spectral bands λ_max_(nm) (DMF)^b^ [Reflectance]
**NBHD** [27]	---	---	---	(375, 306, 266)^**b**^
**1**	3.40	1.63	9.6	[691]
**2**	2.41	1.66	11.7	[652]
**3**	1.87	1.32	2.1	[647]

^a^ Solutions in DMF (10^− 3^ M).

^b^ Concentrated solutions.

### Thermal analysis

The objective of TGA is to successfully verify whether water or ethanol (solvent) molecules exist in the internal or external coordination sphere of the complex^73^.

The first degradation stage of [Cu_4_(L)Cl_6_(H_2_O)_10_].2H_2_O **1** occurs within the range (24–98 °C), which refers to the removal of the two non-coordinated H_2_O molecules (weight loss found/(calcd.%); 3.27/(3.08%). The second great decomposition step (99–270 °C) refers to the removal of the ten coordinated H_2_O, 3Cl_2_, phH, 2C_2_H_2_, N_2_, CO_2_, and ½H_2_ molecules (weight loss found/(calcd.%); 50.96/(50.96%). The third decomposition step (271–478 °C) with weight loss found/(calcd.%); 5.18/(5.30%), is due to loss of 2N_2_ and 3H_2_. Over 478 °C, the ultimate residue is consistent with 4 CuO and C atoms. The metal residue supports formation of tetranuclear complex.

The first decomposition step of [Cu_2_(L)Br_2_(EtOH)_6_] complex **2** extends up to 371 °C corresponding to elimination of six coordinated EtOH and NO_2_ molecules with weight loss found: 30.55% and calc.: 30.68%. The second step (372–598 °C), with weight loss found: 52.90% and calc.: 52.95%, is due to loss of Br_2_, NO_2_, phph, 2C_2_N_2_, phH and ½C_2_H_2_ molecules. The ultimate residue (2CuO and carbon) was noted over 598 °C (found: 16.55% and calc.: 16.30%).

**Fig. 1:**
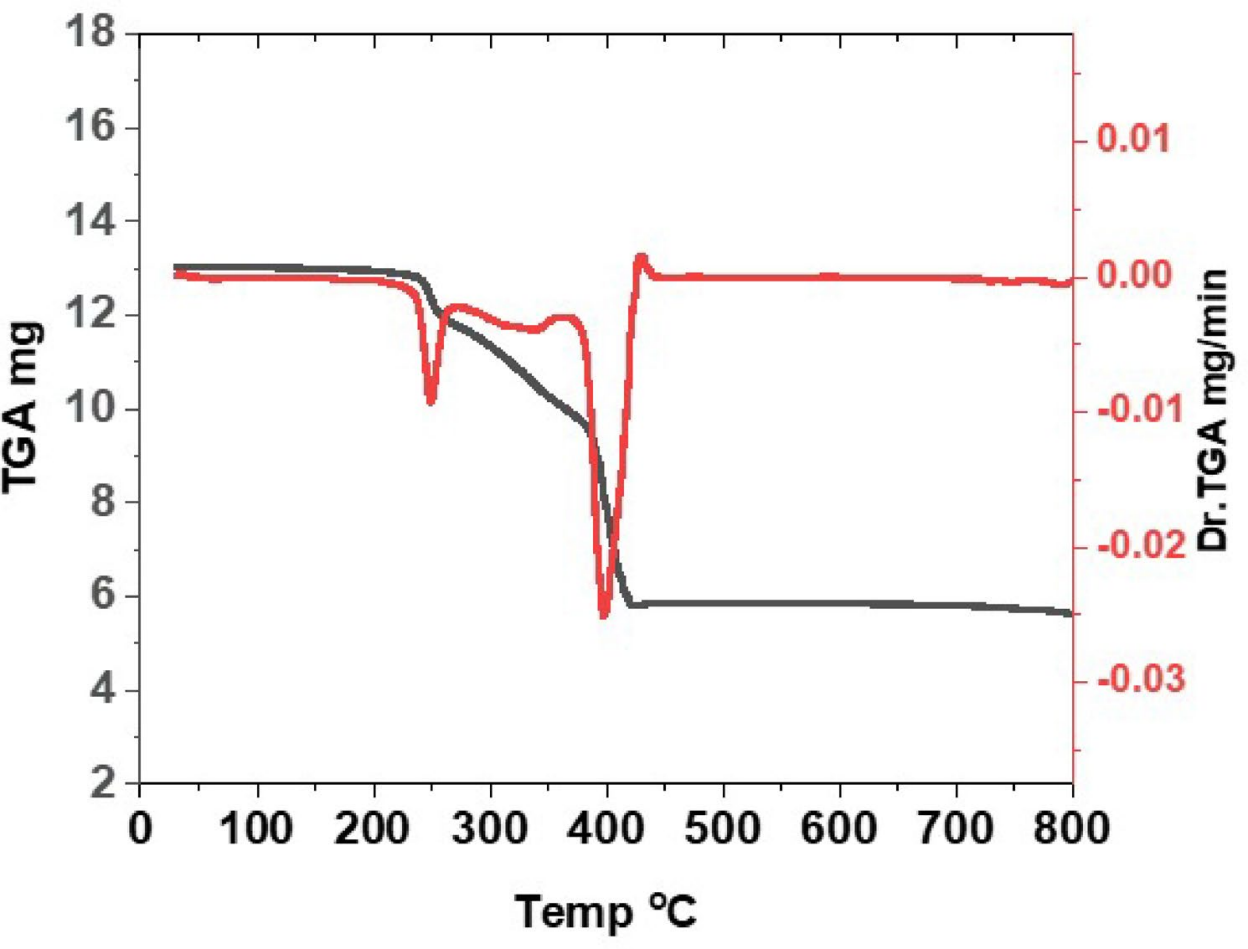
TGA-DTG curve of [Cu_2_(H_2_L)(SO_4_)_2_(H_2_O)_4_] (**3**).

The thermogram of [Cu_2_(H_2_L)(SO_4_)_2_(H_2_O)_4_] **3** (Fig. 1) confirmed the presence of four coordinated water molecules, which were lost up to 262 °C, a weight loss found: 8.53% and calc.: 8.18%. The second stage within the range 263–366 °C, with a weight loss found: 14.85% and calc.: 14.78%, refers to loss of H_2_SO_4_ and O_2_ molecules. The third stage, within the range 367–445 °C, with a weight loss found: 31.58% and calc.: 31.37%, refers to loss of SO_2_, 2C_2_N_2_, 2NO_2_, and 8H_2_ molecules. The ultimate residue “2 CuO and carbon atoms” was detected over 445 °C.

### ESR spectra

**Fig. 2:**
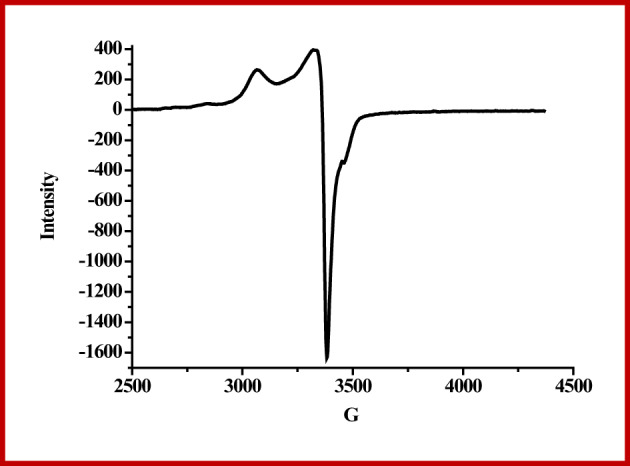
Electron spin resonance spectrum of the sulfato Cu-NBHD complex (**3**).

The electron spin resonance spectrum of Cu-NBHD complex (**3**) is represented in Fig. 2. Table 4 summarizes the spin Hamiltonian parameters. The profile of the spectrum revealed an octahedral geometry^27,74^. This is consistent with electronic spectral data. The spectrum of the Cu-NBHD complex presents an axially *g*-tensor parameters with *g*_||_ > *g*_⊥_ > 2.0023. The covalent nature of copper–NBHD bonds was observed where the gll value of the complex is less than 2.3^75^; *g*_ll_ > 2.3 for ionic nature and < 2.3 for covalent nature. On the other hand, the exchange interaction parameter term “*G”* was calculated^76,77^. The G value is 1.32, demonstrating the presence of copper–copper exchange interactions, for Cu(II)-NBHD complex (**3**), which is in harmony with the fairly low magnetic moment value of the complex (µ_eff_. = 1.32 B.M.; Table 3).

**Table 4:** Recorded electron spin resonance results of Cu(II)-NBHD complex (**3**).

No.	g_II_	g_┴_	g_eff_.	G	A_II_	α^2^	β^2^	γ^2^	K_‖I_	K_┴_	K
**3**	2.0748	2.0568	2.0658	1.3184	258.86	0.8549	0.1979	0.5945	0.4114	0.7129	0.6287

Additionally, molecular orbital coefficients were determined (Table 4)^78^, including α^2^ (covalent the in-plane σ-bonding between the Cu(II) 3 d orbital and NBHD orbitals), β^2^ (covalent in-plane π-bonding), and γ^2^ (covalent out-of-plane σ-bonding). The estimated β^2^ value is smaller than the α^2^ value representing that the in-plane σ-bonding is less covalent than the in-plane π-bonding. The presence of a considerable interaction in the out-of-plane π-bonding is evident from the γ^2^ value^79^. The orbital reduction factor calculation^80^ confirms this also. Covalency was measured using the orbital reduction factor, K, where K < 1 for covalent environments and K = 1 for ionic environments.

It is commonly reported that K_‖_ ≈ K_┴_ ≈ 0.77 for pure σ bonding and that K_‖_ < K_┴_ for in-plane π-bonding; on the other hand, K_┴_ < K_‖_ for out-of-plane π-bonding^77^. According to the data collected, the sulfato Cu-NBHD complex (K = 0.6287) has a covalent character and K_II_ (0.4114) < K_⊥_ (0.7129), which refers to in-plane π-bonding^81^.

**Fig. 3:**
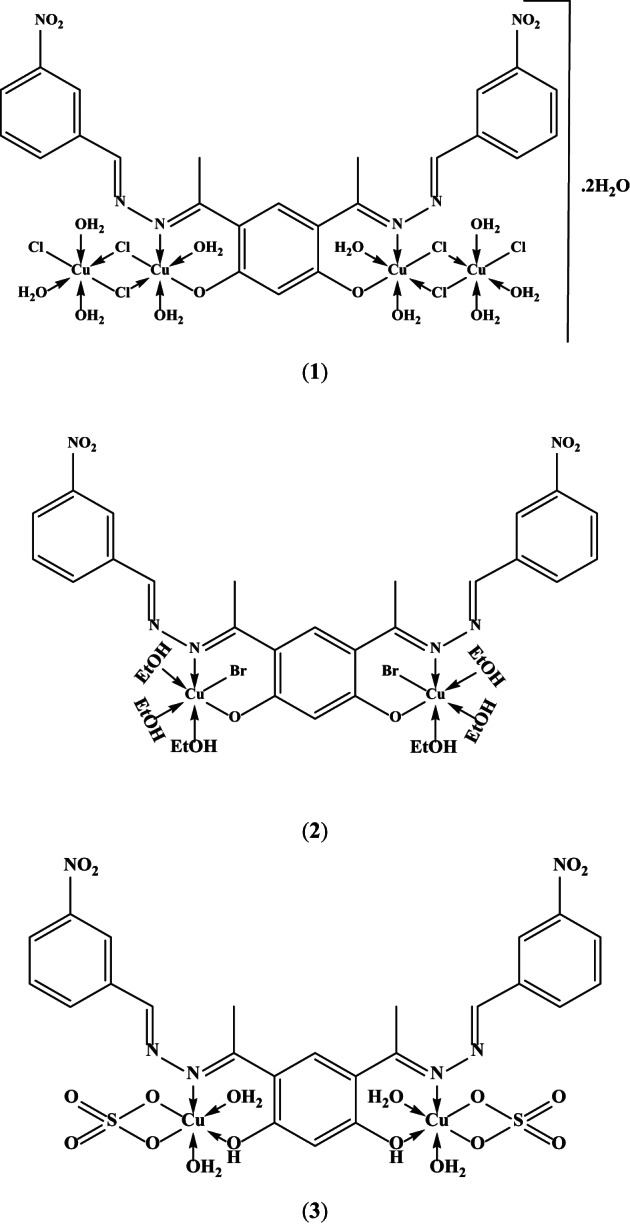
Proposed structures of Cu-NBHD complexes (**1**–**3**).

In light of the above discussion and interpretations, the suggested structures of Cu-NBHD complexes are presented in Fig. 3. The obtained complexes are binuclear complexes in case of bromo **2** and sulfato **3** complexes. However, a tetranuclear complex was obtained in case of chloro complex **1**. Formation of the tetranuclear complex occurs *via* Cl bridging (Fig. 3), similarly occurred in previous published chloro copper(II) complexes^82^.

### XRD measurements

**Fig. 4:**
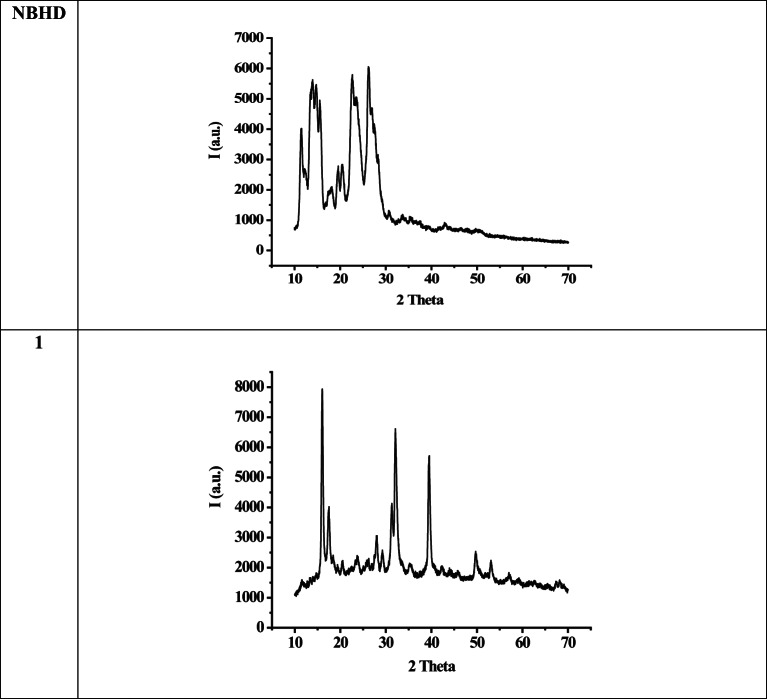
X-ray diffraction of NBHD and Cu-NBHD complex (**1**).

Crystallographic structure for NBHD and Cu-NBHD complexes (**1** & **2**) was examined by using XRD. Figure 4 depicts the XRD pattern of NBHD and the chloro Cu-NBHD complex **1**. It is evident from the patterns that the diffraction angle and intensity in Cu-NBHD complexes differ from those of NBHD. This finding implies formation of Cu-NBHD complexes. Furthermore, the XRD data show that NBHD and Cu-NBHD complexes (**1** & **2**) have a nature that is in between amorphous and crystalline. The average crystallite sizes of the most intense peak for NBHD, **1**, and **2** are 48.6, 232.8, and 459.4 nm, respectively.

### Fluorescence spectral study

Fluorescence spectra of NBHD and Cu-NBHD complexes (**1**–**3**) were measured to determine their emission spectra in a variety of polar solvents. Table 5 shows the measured excitation wavelengths (268, 308, and 306 nm) and maxima emission band in various solvent observed in the range (340–539 = 199, 455–458 = 3, 345–449 = 104, 339–439 = 100 nm) for NBHD ligand, and its Cu-NBHD complexes (**1**–**3**), respectively. This indicates that a red shift of emission band with the increase of solvent polarity purposing n-π*electronic transition [65b]. This interpretation further emphasized by the decreasing of this red shift to about half of the free ligand (199 nm) upon coordination of the non-bonding electrons of the coordinating centers of free ligand to the Cu(II) ion (100 nm); except the chloro-complex that has a little red shift as shown in Table 5. Furthermore, the obvious decrease in the emission intensity referred to the quenching which might mainly arise from the paramagnetic properties of the Cu(II) ion in the complexes. According to the mentioned data, NBHD ligand has the best optical characteristics and well applicants in the solar cell devices than the Cu-NBHD complexes.

The Stoke’s shifts of NBHD, and Cu-NBHD complexes (**1**–**3**) in various polar solvents are listed in Table 5, which shifted as the polarity of solvent increases (7902–18761, 10538–10633, 3694–10408, and 3181–9901 cm^− 1^, respectively). In polar conditions, this was associated with variations in the dipole moment concerning the ground and excited states^10^.

**Table 5:** Excitation and emission spectral data of NBHD and Cu-NBHD complexes (**1**–**3**) in different solvents at room temperature.

Solvent	NBHD λ_ex_ = 268/nm		1 λ_ex_ = 308/nm		2 λ_ex_ = 306/nm		3 λ_ex_ = 306/nm	
	λ_em_	(νa-νf)/cm^− 1^	λ_em_	(νa-νf)/cm^− 1^	λ_em_	(νa-νf)/cm^− 1^	λ_em_	(νa-νf)/cm^− 1^
**1**,**4-dioxane**	340	7902	457	10,586	350	4108	355	4511
**benzene**	347	8495	456	10,538	345	3694	343	3525
**ether**	438	14,482	456	10,538	346	3778	339	3181
**isopropanol**	----	----	457	10,586	437	9796	430	9424
**ethanol**	365	9916	458	10,633	434	9638	408	8170
**methanol**	----	----	457	10,586	438	9849	429	9370
**chloroform**	----	----	457	10,586	----	----	----	----
**DMF**	539	18,761	455	10,490	442	10,055	----	----
**ethyl acetate**	364	9841	458	10,633	449	10,408	439	9901
**acetone**	359	9458	455	10,490	431	9478	364	5207
**1**,**2- dichloroethane**	344	8244	458	10,633	347	3861	350	4108

### DFT calculations

**Fig. 5:**
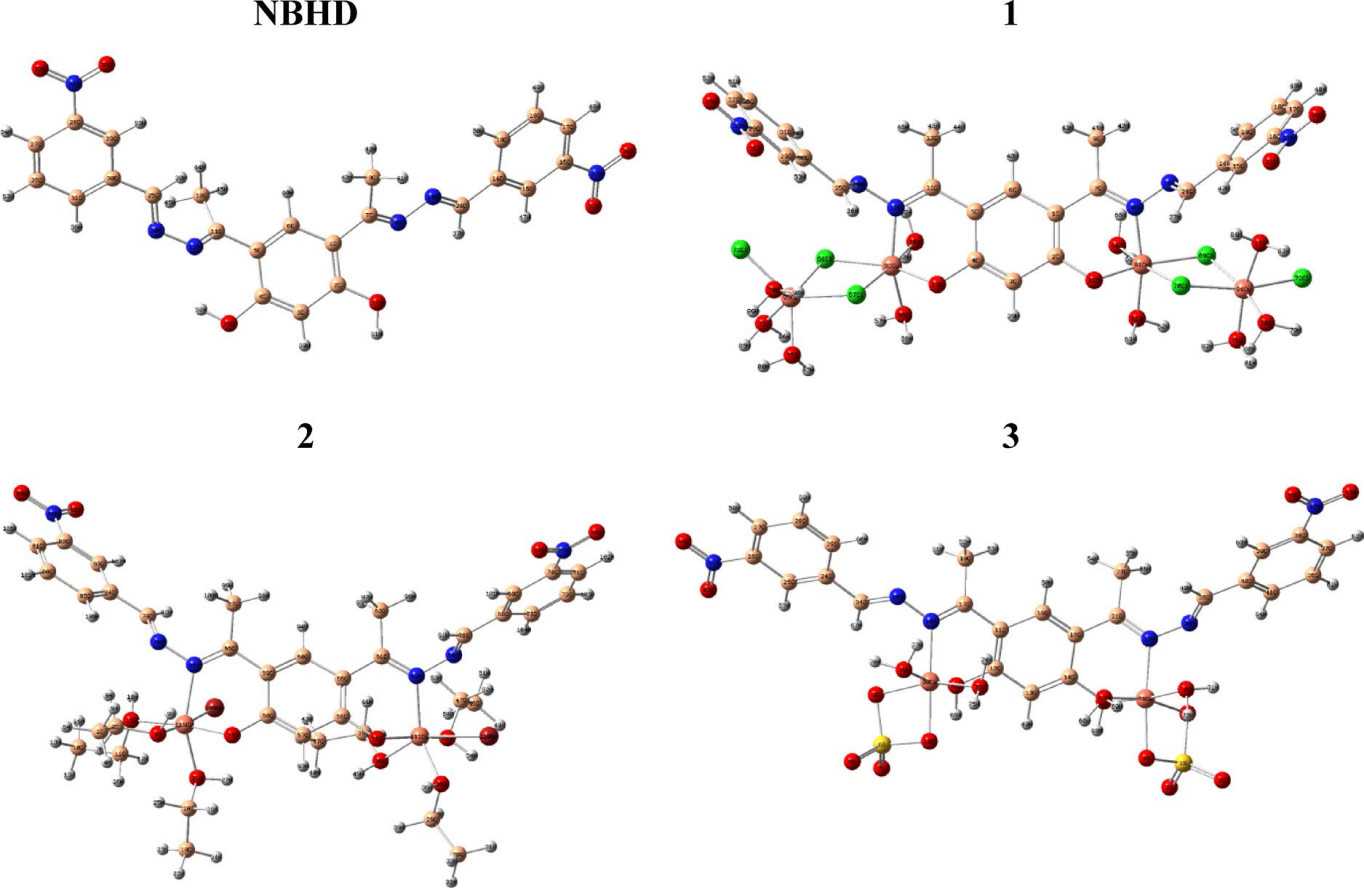
Optimized 3D structures of NBHD and Cu-NBHD complexes (**1–3**).

The molecular modelling of NBHD and Cu-NBHD complexes (**1**–**3**) was efficiently performed using DFT calculations using B3LYP coupled with 6-311G(d, p) and LanL2dz for (C, H, N, O, S, Cl, and Br), and metal atoms, respectively (Fig. 5). Also, E_HOMO_, E_LUMO_, energy gap (ΔE), and other essential parameters were evaluated, Table 6.

**Fig. 6:**
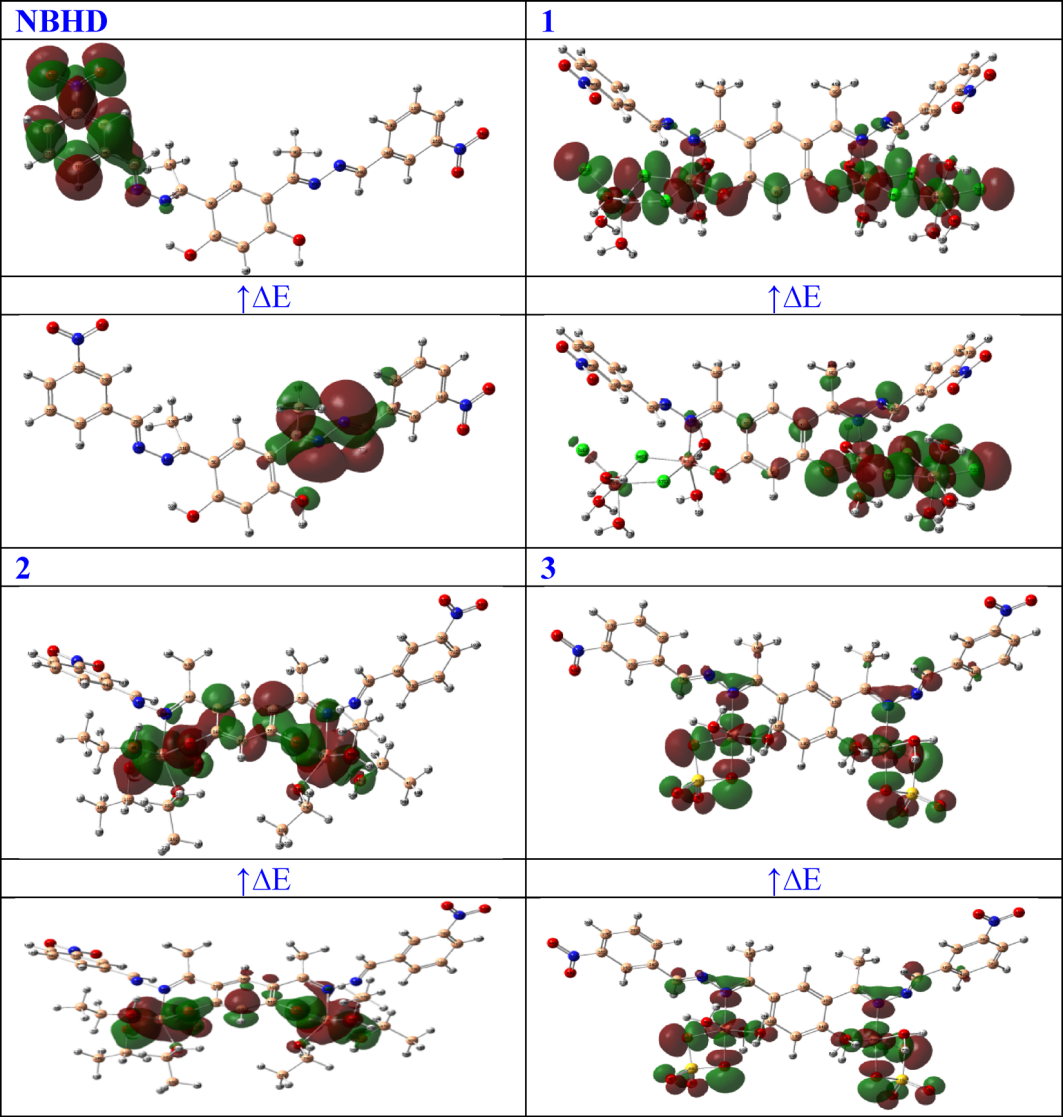
HOMO-LUMO distribution of NBHD and Cu-NBHD complexes (**1–3**).

The HOMO and LUMO energies of NBHD and Cu-NBHD complexes (**1–3**) are shown in Table 6. Figure 6 shows their HOMO-LUMO distribution and their + ve and -ve regions. The positive and negative phases are colored green and red, respectively.

A higher (less negative) E_HOMO_ value clearly suggests a stronger capability to donate electrons^83^. For NBHD, the E_HOMO_ value is −6.45 eV. Comparatively, the bromo complex **2** has a slightly higher E_HOMO_ value of −6.16 eV, chloro complex **1** has an E_HOMO_ of −6.22 eV, and sulfato complex **3** has the highest E_HOMO_ value of −5.83 eV among the complexes. A lower (more negative) E_LUMO_ value signifies a stronger ability to accept electrons^84^. The E_LUMO_ for NBHD is −2.93 eV, which is less negative than the E_LUMO_ values of the complexes: −3.46 eV for the chloro complex **1**, −3.65 eV for the bromo complex **2**, and − 3.52 eV for the sulfato complex **3**. The energy gap “ΔE” is the difference between “E_HOMO_” and “E_LUMO_” and is an indicator of the molecule’s chemical reactivity^85^. A smaller energy gap evidently suggests higher reactivity and lower stability. The energy gap for NBHD is 3.53 eV. Complexes **1**, **2**, and **3** have smaller energy gaps of 2.76 eV, 2.51 eV, and 2.30 eV, respectively (Fig. S4; Supplementary materials). This indicates that the complexes are more reactive than NBHD. A small ionization potential “IP” implies easier electron removal, thus higher reactivity. The IP for NBHD is 6.45 eV. The bromo complex **2** has a lower IP of 6.16 eV, chloro complex **1** has an IP of 6.22 eV, and sulfato complex **3** has the lowest IP of 5.83 eV. The electron affinity (EA) measures the ability of a molecule to accept an electron^86^. A higher EA indicates a greater tendency to accept electrons. The EA for NBHD is 2.93 eV. In comparison, the bromo complex **2** has the highest EA of 3.65 eV, the chloro complex **1** has an EA of 3.46 eV, and the sulfato complex **3** has an EA of 3.52 eV.

Electronegativity (χ) is a measure of the tendency of an atom or molecule to attract electrons towards itself^87^. The electronegativity of NBHD is 4.69 eV. For the complexes, the values are 4.84 eV for complex **1**, 4.91 eV for complex **2**, and 4.67 eV for complex **3**. The bromo complex **2** exhibits the highest electronegativity, revealing a stronger tendency to attract electrons compared to the NBHD and the other complexes. The sulfato complex **3**, with slightly lower electronegativity than NBHD, suggests a somewhat reduced ability to attract electrons. Chemical potential (µ) indicates the tendency of a molecule to escape from its chemical environment. A more negative chemical potential implies greater stability^88^. The chemical potential for NBHD is −4.69 eV. Complexes **1**, **2**, and **3** have chemical potentials of −4.84 eV, −4.91 eV, and − 4.67 eV, respectively. This indicates that chloro **1** and bromo **2** complexes are more stable than NBHD, with the bromo complex **2** being the most stable. Chemical hardness (η) of a molecule indicates its resistance to alterations in its electron distribution. Large hardness value indicates greater stability and lower reactivity. The chemical hardness of NBHD is 1.76 eV. The hardness values for the complexes are 1.38 eV for complex **1**, 1.26 eV for complex **2**, and 1.15 eV for complex **3**. This indicates that the metal complexes are softer and therefore more reactive compared to the free ligand. Softness (σ) is the reciprocal of hardness and is a measure of the reactivity of a molecule. A higher softness value indicates higher reactivity. The softness for NBHD is 0.28 eV^1-^. The complexes have softness values of 0.36 eV^−1^ for complex **1**, 0.40 eV^−1^ for complex **2**, and 0.43 eV^−1^ for complex **3**. This confirms that the metal complexes are more reactive than NBHD. A molecule’s capacity to receive electrons is gauged by its electrophilicity (ω). A higher electrophilicity index indicates a greater tendency to accept electrons and thus greater reactivity towards nucleophiles. The electrophilicity index for NBHD is 6.24 eV. The bromo complex **2** has a significantly higher value of 9.58 eV, the chloro complex **1** has 8.49 eV, and the sulfato complex **3** has 9.49 eV. This shows that the metal complexes are much more electrophilic than the free ligand. Nucleophilicity (Nu) measures the ability of a molecule to donate electrons. A higher nucleophilicity value indicates a greater tendency to donate electrons. The nucleophilicity for NBHD is 0.16 eV. In comparison, the bromo complex **2** has a lower value of 0.10 eV, the chloro complex **1** has 0.12 eV, and the sulfato complex **3** has 0.11 eV.

In summary, the coordination of NBHD with metals in complexes **1**, **2**, and **3** generally enhances their reactivity compared to the free NBHD, as indicated by the trends in all calculated parameters.

**Table 6:** Important calculated parameters of NBHD and Cu-NBHD complexes (**1–3**).

Compound	E_HOMO_ eV	E_LUMO_ eV	∆E eV	IP eV	EA eV	χ eV	µ eV	η eV	σ eV^− 1^	ω eV	Nu eV
**NBHD**	−6.45	−2.93	3.53	6.45	2.93	4.69	−4.69	1.76	0.28	6.24	0.16
**1**	−6.22	−3.46	2.76	6.22	3.46	4.84	−4.84	1.38	0.36	8.49	0.12
**2**	−6.16	−3.65	2.51	6.16	3.65	4.91	−4.91	1.26	0.40	9.58	0.10
**3**	−5.83	−3.52	2.30	5.83	3.52	4.67	−4.67	1.15	0.43	9.49	0.11

Recognizing the distribution of fractional charges on substrates and proteins is essential for predicting their interactions during molecular docking. Molecular electrostatic potential (MEP) drawings are valuable tools that visually represent the structural and topological characteristics of molecules in three dimensions. These diagrams use a color gradient, where blue indicates positively charged regions and red indicates negatively charged regions, to illustrate the MEP^89,90^. The blue areas correspond to regions of electrophilic reactivity, while the red areas correspond to nucleophilic reactivity. This color coding provides critical perceptions into the electrostatic characters and potential interaction sites between substrates and proteins during docking^91^.

**Fig. 7:**
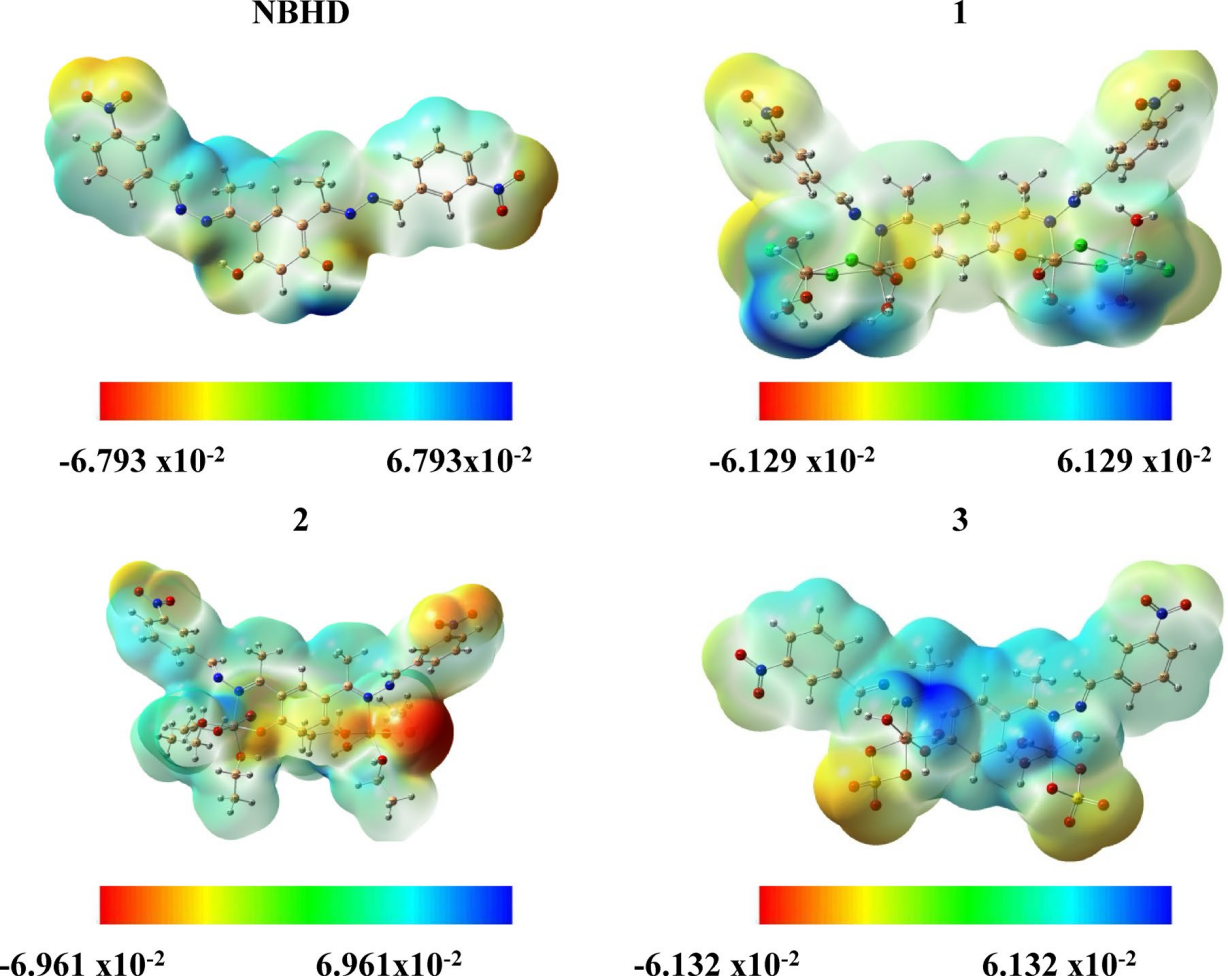
Molecular electrostatic potential (MEP) map of NBHD and Cu-NBHD complexes (**1**–**3**).

The MEP diagram, established from the optimized geometry of NBHD and Cu-NBHD complexes and generated using the same theoretical approach, is shown in Fig. 7. In this diagram, red regions around heteroatoms indicate electron-rich areas, which are likely targets for electrophilic attacks. Conversely, blue regions, often surrounded by hydrogens, suggest potential sites for intermolecular interactions between proteins and substrates. This visualization helps to understand the reactivity and interaction potential of different molecular regions.

### Antitumor effect and molecular Docking studies

Nowadays, pharmacological scientists are looking for many effective cancer-curing drugs. The manufacture of metal-derived anticancer medications is one of the most important strategies for solving this issue. As a result, many transition metal complexes have been developed and their potential for cancer treatment have been investigated^92,93^.

The third most common cause of cancer-related death globally is liver cancer. Liver cancer is a serious public health issue since it has such a profound effect on our lives. One common hepatic cell line is HepG-2. It is employed in numerous investigations, ranging from the cytotoxicity of compounds on the liver to oncogenesis^94^.

**Fig. 8:**
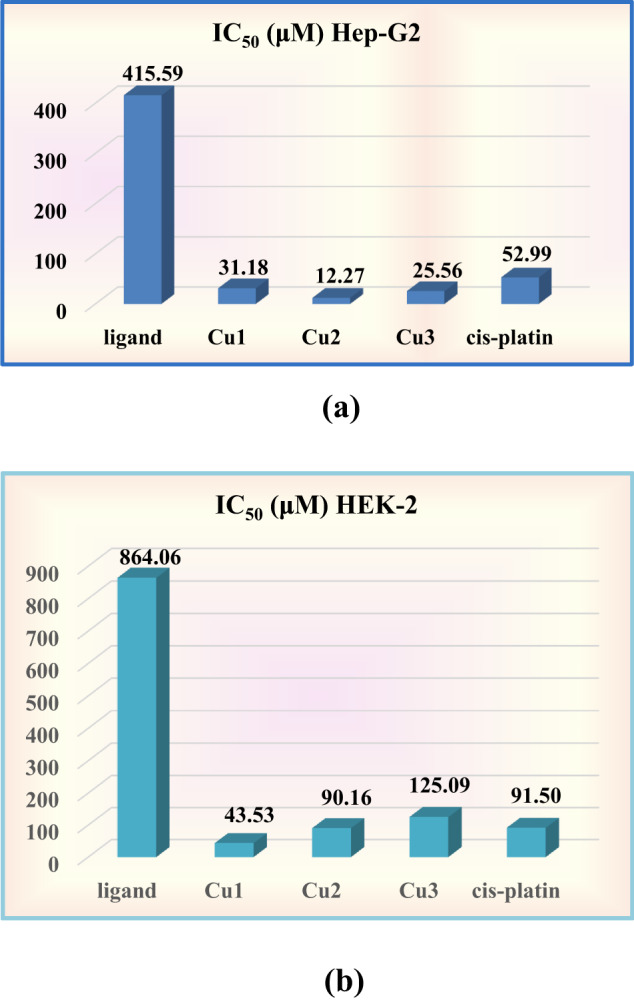
(**a**) IC_50_ for NBHD, Cu-NBHD complexes (**1**–**3**), and *cis*-Pt against HepG-2. (**b.**)IC_50_ for NBHD, Cu-NBHD complexes (**1**–**3**), and *cis*-Pt against HEK-2.

Table 7 and Fig. 8 summarize and illustrate the *in vitro* anticancer activity of Cu-NBHD complexes against the human Hepatocellular carcinoma cell line (HepG-2) and the normal Human embryonic kidney cells (HEK-2). Towards HepG-2, the antitumor activity measured by IC_50_/µM of free NBHD ligand (415.59 µM) exhibited a pronounced improvement through complexation with various salts of copper(II) to be 31.18, 12.27, and 25.56 µM for Cu(II) complexes (**1**–**3**), respectively. The results obtained agree with previous findings for analogous complexes of hydrazone ligands^27,28,49^. This finding is directly proportional with the stability of the current complexes as indicated from the + ve slope of IC_50_ of HepG-2 *versus* ∆E (analogous to stability); IC_50_ HepG-2 (µM) = − 833.36 + 343.97∆E, R² = 0.89, *n* = 4; whereas, in case of HEK-2 showed an opposite trend (-ve slope),: IC_50_ HEK-2 (µM) = 534.35-177.58 ∆E, R² = 0.9989, *n* = 3. As we seen from the slope’s values of HepG-2 and HEK-2, it was observed that the stability factor (∆E) enhanced the antitumor activity (IC_50_). The greater effect of the complexes compared to NBHD may be explained by the improved conjugation in the NBHD framework because of coordination with Cu(II) ion^95^. An increase in NBHD’s π-electron delocalization facilitates the complexes’ penetration through the cell membrane’s lipid coat^96^. Moreover, the significant function that copper plays in a diversity of enzymes that catalyze a varied processes may be the cause of the copper (II) complex’s excellent activity^97,98^.

It was noted that the bromo complex **2** exhibited the highest activity (IC_50_ = 12.27 ± 0.37 µM). While the lowest activity is provided by the chloro complex (IC_50_**=** 31.18 ± 1.40 µM). This trend is consistent with the previously published work including copper hydrazone complexes^99^. Furthermore, variation of the activity given by the complexes (Br^−^SO_4_^2−^Cl^−^) indicates the effect of anion upon activity as reported for copper hydrazone complexes^31,100^. The bromo complex (**2**) has a lower IC_50_ (12.27 ± 0.37 µM) than that of *cis*-platin (IC_50_ = 52.99 ± 1.67 µM). Additionally, the cytotoxicity properties of NBHD and copper-NBHD complexes were assessed using normal HEK-2 cells. Interestingly, the bromo complex **2** showed a higher IC_50_ value (90.16 µM), which is approximately the same IC_50_ value given by *cis*-platin (IC_50_ = 91.5 µM). Thus, the bromo complex **2** is an encouraging substance that may be investigated further. Its mechanism of action and possible synergistic effects when taken with other chemotherapeutic drugs might be investigated in more detail. This may result in better treatment approaches for cancer types that are resistant to treatment.

**Table 7:** Anticancer effect of NBHD and Cu-NBHD complexes (**1**–**3**) against HepG-2 and HEK-2 cell lines.

No.	Compound	IC_50_ (µM)^a^	
		HepG-2	HEK-2
	NBHD	415.59 ± 14.39	864.06 ± 13.70
**1**	[Cu_4_(L)Cl_6_(H_2_O)_10_].2H_2_O	31.18 ± 1.40	43.53 ± 2.00
**2**	[Cu_2_(L)Br_2_(EtOH)_6_]	12.27 ± 0.37	90.16 ± 6.32
**3**	[Cu_2_(H_2_L)(SO_4_)_2_(H_2_O)_4_]	25.56 ± 1.11	125.09 ± 4.25
	*Cis-platin*	52.99 ± 1.67	91.50 ± 2.62

^a^ IC_50_ values are the mean ± SD of three replicates.

Identifying the chemicals’ inhibitory potential and forecasting how they will attach to the mobility in the enzyme pocket are two things that molecular docking studies can assist with. Computational drug design heavily relies on molecular docking techniques^84^. Estimating the interaction between synthesized chemicals and enzyme receptors is a common application of this computational approach. It is helpful to identify the ideal binding configurations as well as variables that lead to the novel complex having the smallest overall energy^85^.

We started by re-docking the native ligand, N-1-[cis-3-(acetylamino)cyclobutyl]−1 H-imidazol-4-yl-2-(4-methoxyphenyl)acetamide, which was co-crystallized with the CDK-2 active site, in order to validate the molecular docking technique. The docking method’s applicability for the present study was demonstrated by the fruitful replication of the binding pattern seen with the co-crystallized ligand. As shown in Fig. S5. (supplementary materials), the re-docked ligand showed a close alignment with the native co-crystallized ligand. As demonstrated in Fig. S5 (supplementary materials), the H-bonds that were created between the docked ligand and the 3IG7 amino acids (LEU 83, GLU 81, and LYS 33) closely matched those that were generated by the native ligand, further confirming the reliability of our docking simulations.

**Fig. 9:**
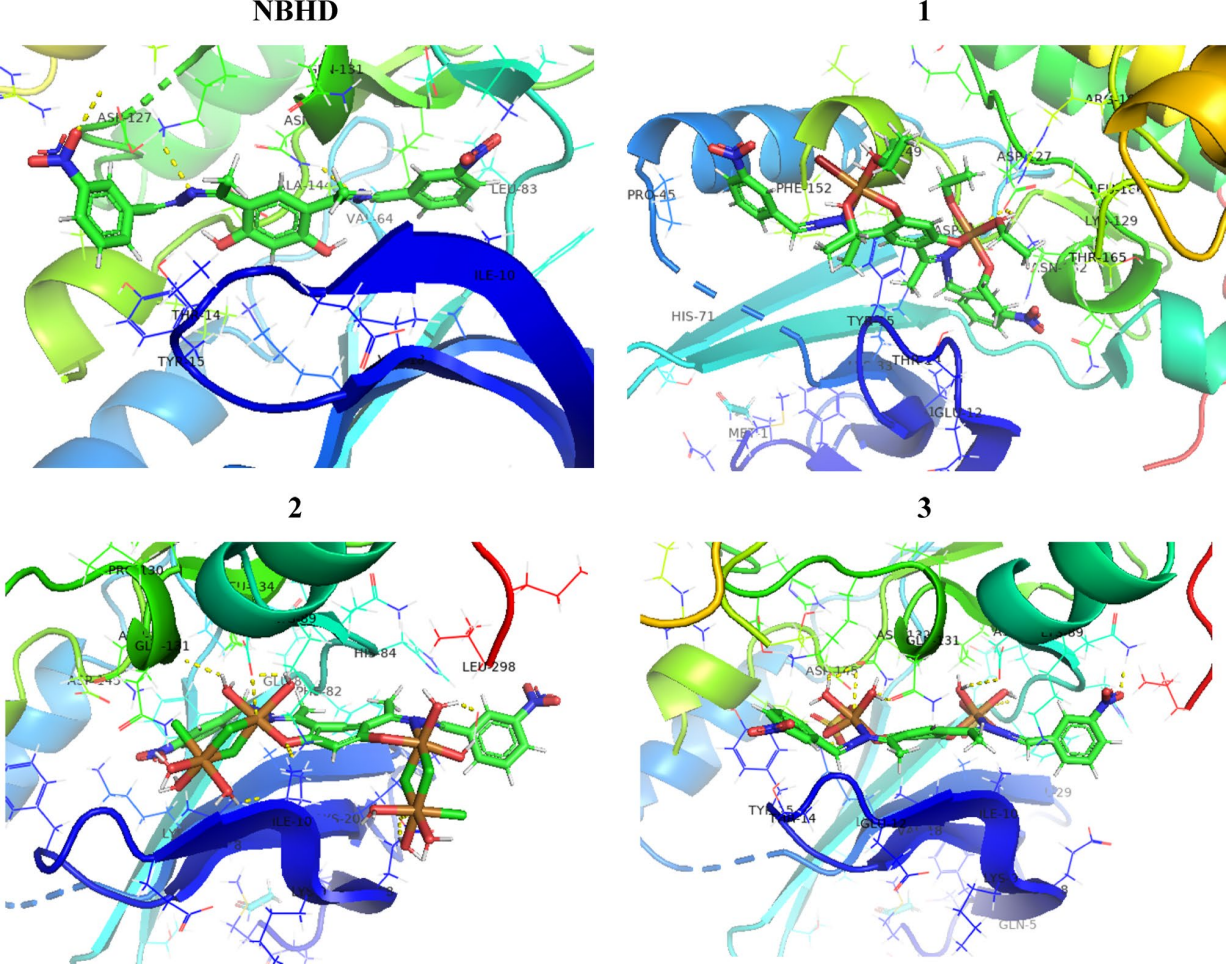
3D of docked compounds through the 3IG7.

The molecular docking was performed by AutoDock and the best binding mode with docked compounds are shown in Fig. 9 and Fig. S6; Supplementary materials), while the interactions with amino acids residue were shown in Table 8. NBHD ligand interacts with the target protein through hydrogen bonds with several key amino acid residues: ASP 127, LYS 89, ARG 169, and THR 165. The binding affinity (ΛG) for this interaction is −6.49 kcal/mol, indicating a moderate level of binding strength. The chloro complex **1** exhibits a binding affinity (ΛG) of −8.18 kcal/mol, with a notable array of hydrogen bonds formed with residues GLN 131, ASP 86, LEU 298, GLU 8, and ILE 10. The interaction with GLN 131 involves polar side chains, forming hydrogen bonds that enhance binding stability. The bromo complex **2** shows an improved binding affinity (ΛG) of −8.50 kcal/mol, indicating a stronger interaction with the target protein compared to NBHD. This enhanced affinity is due to hydrogen bonds with residues GLY 13, ASP 127, ASP 145, ASN 132, and LYS 129. The sulfato complex **3** shows the highest binding affinity (ΛG) of −8.95 kcal/mol, indicating the strongest interaction with the target protein. The complex forms hydrogen bonds with a wide range of residues, including GLN 131, ASN 132, ASP 145, ASP 86, GLY 13, LYS 33, LEU 83, GLN 85, and THR 165. In summary, complex-formation greatly increases binding affinity with the target protein when NBHD and Cu-NBHD complexes (**1**, **2**, and **3**) are compared. More persistent and energetically advantageous ligand-protein interactions are the consequence of the metal complexes’ formation of numerous, powerful hydrogen bonds with different amino acid residues.

**Table 8:** Molecular Docking of NBHD and Cu-NBHD complexes with the target protein (3IG7).

compound	Amino Acids residue	Binding affinity (ΛG) (kcal/mol)
**NBHD**	ASP 127, LYS 89, ARG 169, THR 165	−6.49
**1**	GLN 131, ASP 86, LEU 298, LEU 298, ASP 86, GLU 8, ILE 10, GLN 131, GLU 8	−8.18
**2**	GLY 13, ASP 127, ASP 145, ASN 132, LYS 129	−8.50
**3**	GLN 131, ASN 132, ASN 132, ASP 145, ASP 86, GLY 13, LYS 33, LEU 83, GLN 85, THR 165	−8.95

## Conclusion

Reactions of the symmetrical hydrazone ligand (NBHD) with different copper(II) salts; chloride, bromide, and sulfate yielded new copper(II) complexes, which were characterized by various techniques. Binuclear complexes were obtained in case of bromide and sulfate anions, while a tetranuclear complex with Cl bridging was obtained in case of chloride ion. Molar conductivity indicated neutral characters for all Cu-NBHD complexes. The FT-IR spectral data revealed that coordinating of NBHD occurs *via* phenolic oxygen and azomethine nitrogen atoms. NBHD acts as a *bis*(monobasic bidentate) in case of Cl^−^ and Br^−^ ions and *bis*(neutral bidentate) in case of SO_4_^2−^ ion. Magnetic moment, electronic and ESR spectra demonstrated distorted octahedral geometrical structures. To study the solvatochromic behavior of NBHD and its Cu(II) complexes, fluorescence spectra have been studied in a variety of solvents. Molecular modeling of NBHD and its Cu-NBHD complexes was carried out. Cu-NBHD complexes exhibited anticancer action against hepatocellular carcinoma, which is correlated with molecular docking simulations. The bromo complex **2** showed an enhanced activity than that of *cis*Pt. The promising activity prompts further studies about the complex as an antitumor agent.

## Supplementary Information

Below is the link to the electronic supplementary material.

Supplementary Material 1

## Data Availability

The data that support the findings of this study are available from the corresponding author upon reasonable request.
