# Supplementary material for: Tailoring, spectroscopic, DFT, solvatochromic, antitumor, and molecular docking studies of new polynuclear Cu(II)-hydrazone complexes
Source: Sci Rep. 2026 Jan 6;16:795. doi: 10.1038/s41598-025-32666-8 (PMC12780247; doi:10.1038/s41598-025-32666-8)
Supplement: Supplementary file 1 — Supplementary Material 1 [file 41598_2025_32666_MOESM1_ESM.docx]

**Supplementary materials**

Elemental analyses (%) (C, H and N) of Cu-NBHD complexes were estimated in the Ministry of Defense, Chemical War Department. (% M) in Cu-NBHD complexes was determined by Na_2_EDTA complexometrically after degradation of the chelates by using conc. HNO_3_. A digital Stuart *SMP3* melting point apparatus” three samples” was used to record the decomposition temperatures of Cu-NBHD complexes. IR spectra of Cu-NBHD complexes were recorded in potassium bromide pellets on a Nicolet 6700 FT IR spectrometer (4000-400 cm^-1^). The electron spin resonance spectrum of powdered sample of Cu-NBHD complexes was recorded on an Elexsys (E500), Bruker company instrument and DPPH (2,2′-diphenyl-1-picrylhydrazyl) was used as a reference material. UV-Vis. spectral measurements of Cu-NBHD complexes were carried out as solutions in dimethylformamide and/or Nujoll mulls on “V-550” Jasco UV-Vis. spectrophotometer. A Johnson Matthey magnetic susceptibility balance (Alfa product; MKI) was used to measure the magnetic susceptibility of the Cu-NBHD complexes. The obtained effective magnetic moment values were corrected using Pascal's constants for the diamagnetism of all atoms included in the chelates. Thermal gravimetric analysis is carried out on a Shimadzu-50 instrument (under N2 and at a heating rate = 10 ^o^C/min). The corning conductivity meter “NY 12631 model 441” was used to record the molar conductivity values of complexes (1x10^-3^ M) solutions. The fluorescence spectral data of Cu-NBHD compounds were obtained using a Perkin Elmer LS 55 Luminescence Spectrometer (USA) (Ain Shams University, Cairo, Egypt).

***2.5. Anticancer activity***

***2.5.1. Cell line Propagation***

50 µg/mL Gentamycin and 10% deactivated fetal calf serum were added to the RPMI-1640 medium used to cultivate the cells. Two to three times a week, the cells were sub-cultured at 37 ºC in a humidified environment with 5% CO_2_.

***2.5.2. Cytotoxicity assessment using viability assay***

For antitumor assays, the tumor cell lines were suspended in medium at concentration 5x104 cell/well in Corning® 96-well tissue culture plates, then incubated for 24 hr. The tested compounds were then added into 96-well plates (three replicates) to achieve eight concentrations for each compound. Six vehicle controls with media or 0.5 % DMSO were run for each 96 well plate as a control. After incubating for 24 h, the numbers of viable cells were determined by the MTT test. Briefly, the media was removed from the 96 well plate and replaced with 100 µl of fresh culture RPMI 1640 medium without phenol red then 10 µl of the 12 mM MTT stock solution (5 mg of MTT in 1 mL of PBS) to each well including the untreated controls. The 96 well plates were then incubated at 37°C and 5% CO_2_ for 4 hours. An 85 µl aliquot of the media was removed from the wells, and 50 µl of DMSO was added to each well and mixed thoroughly with the pipette and incubated at 37°C for 10 min. Then, the optical density was measured at 590 nm with the microplate reader (SunRise, TECAN, Inc, USA) to determine the number of viable cells and the percentage of viability was calculated as [(ODt/ODc)]x100% where ODt is the mean optical density of wells treated with the tested sample and ODc is the mean optical density of untreated cells. The relation between surviving cells and drug concentration is plotted to get the survival curve of each tumor cell line after treatment with the specified compound. The 50% inhibitory concentration (IC_50_), the concentration required to cause toxic effects in 50% of intact cells, was estimated from graphic plots of the dose response curve for each conc. using Graphpad Prism software (San Diego, CA. USA).


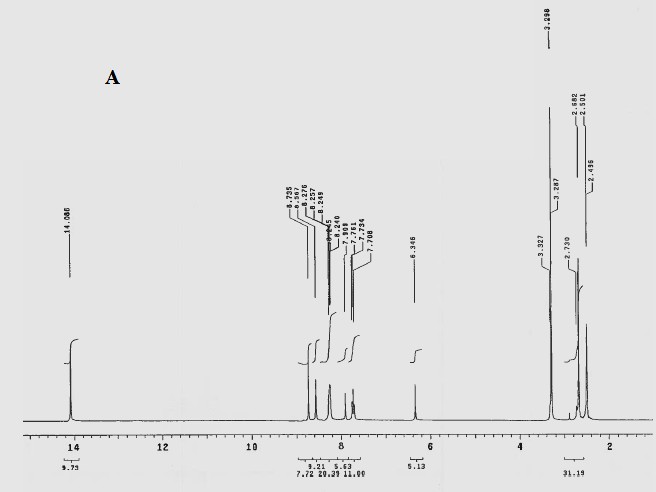

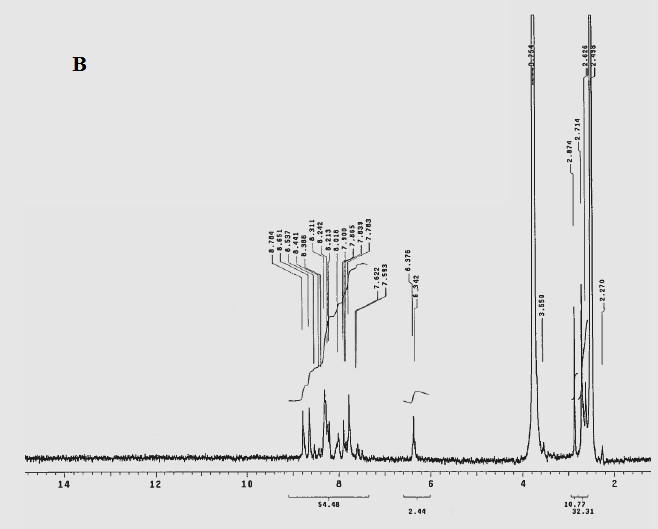


**Fig. S1.** ^1^H NMR spectra of the ligand relative to TMS **A)** in DMSO*-d6* & **B)** in addition of D_2_O.

| **NBHD** | **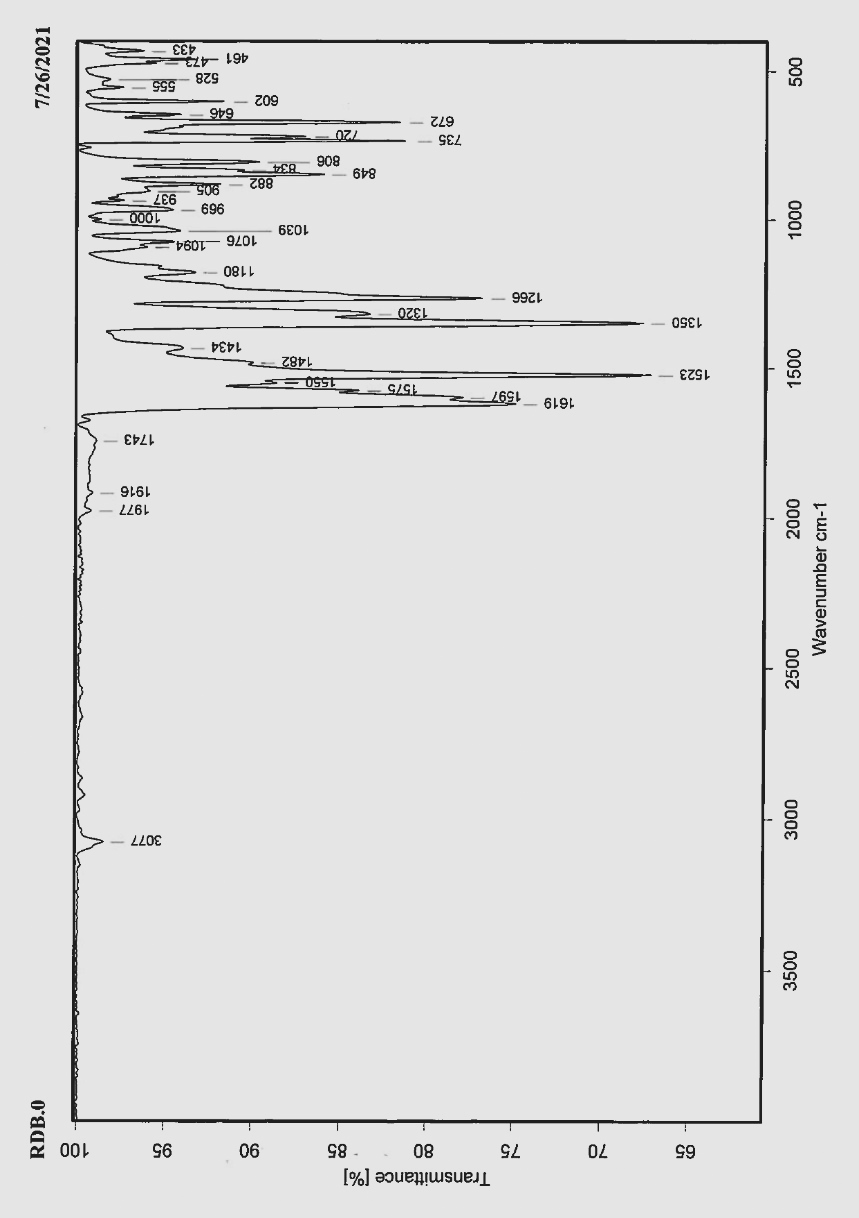** |
| --- | --- |
| **1** | **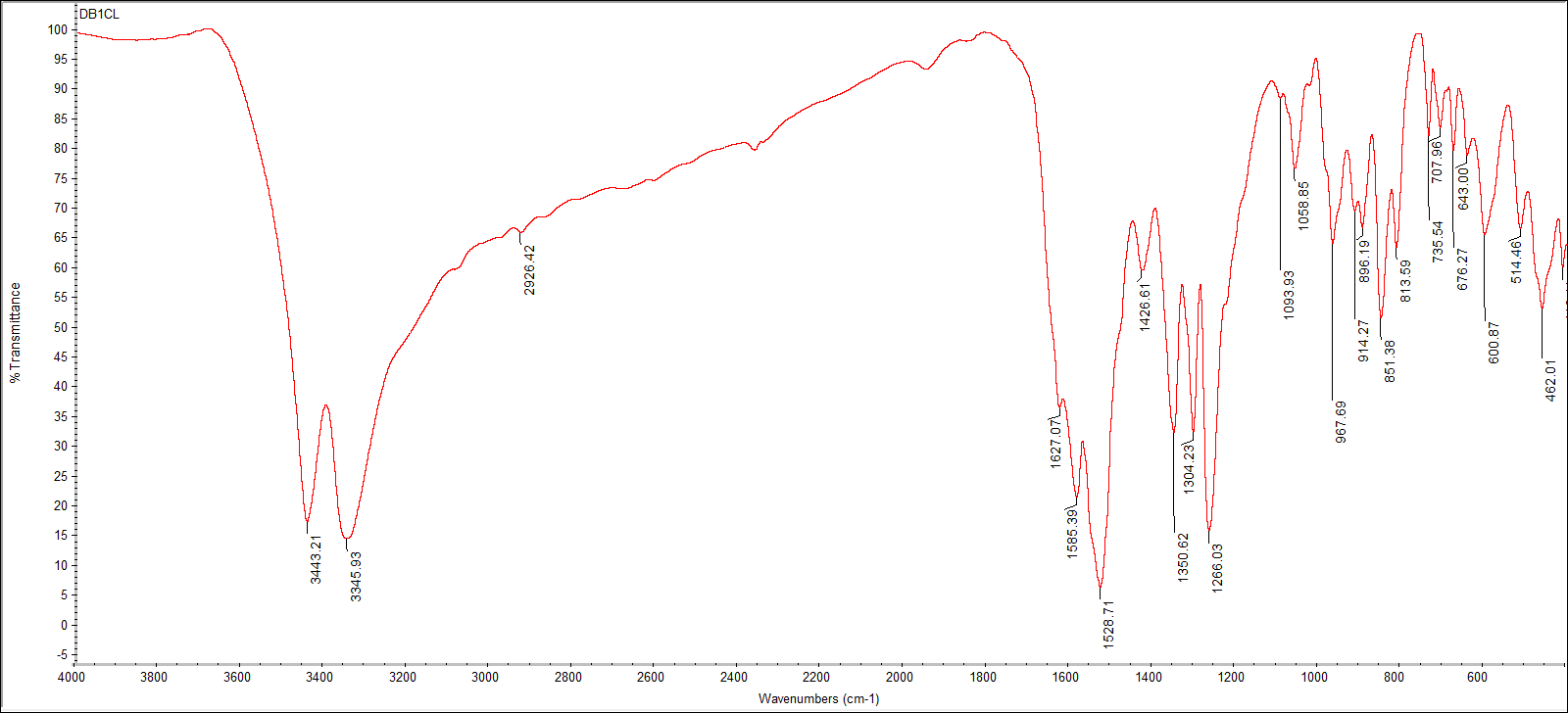** |
| **2** | **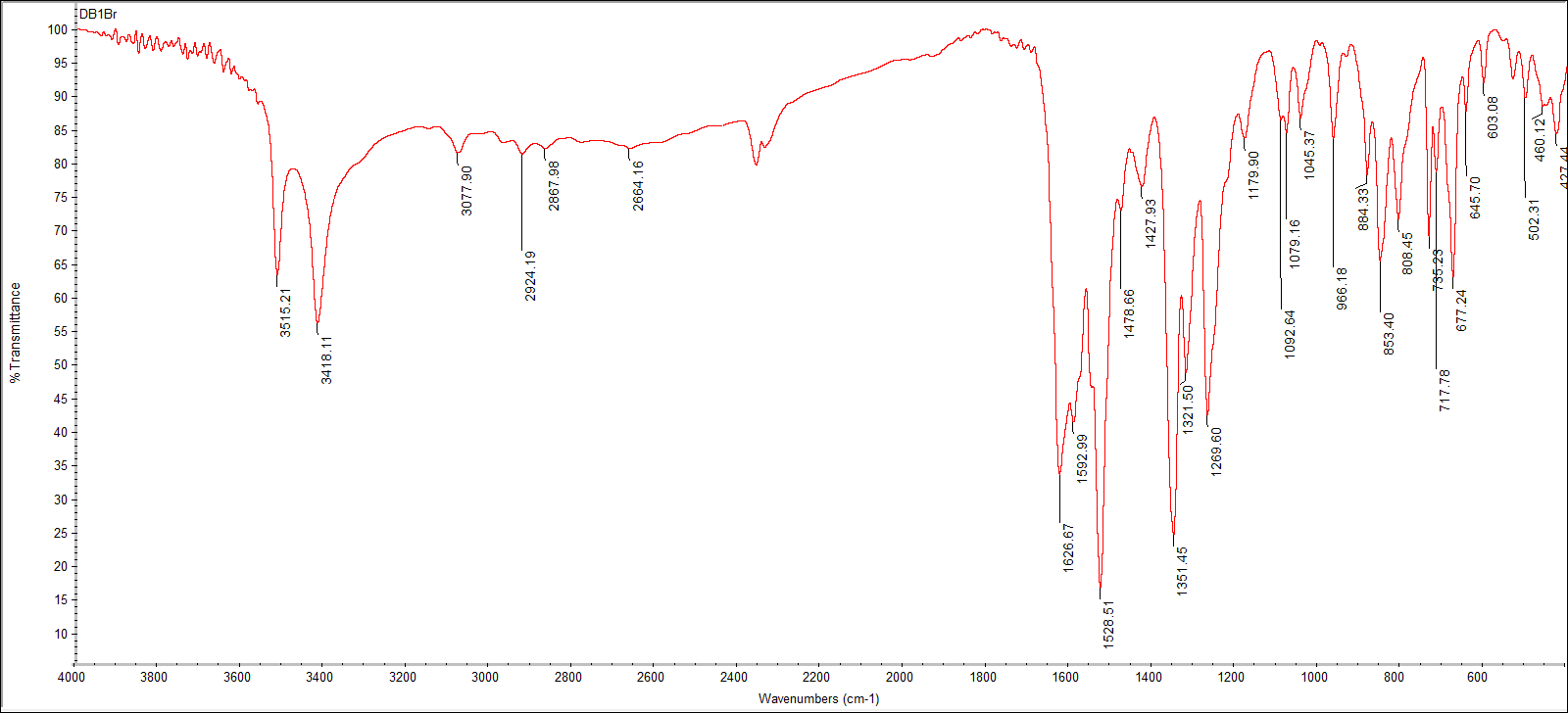** |
| **3** | **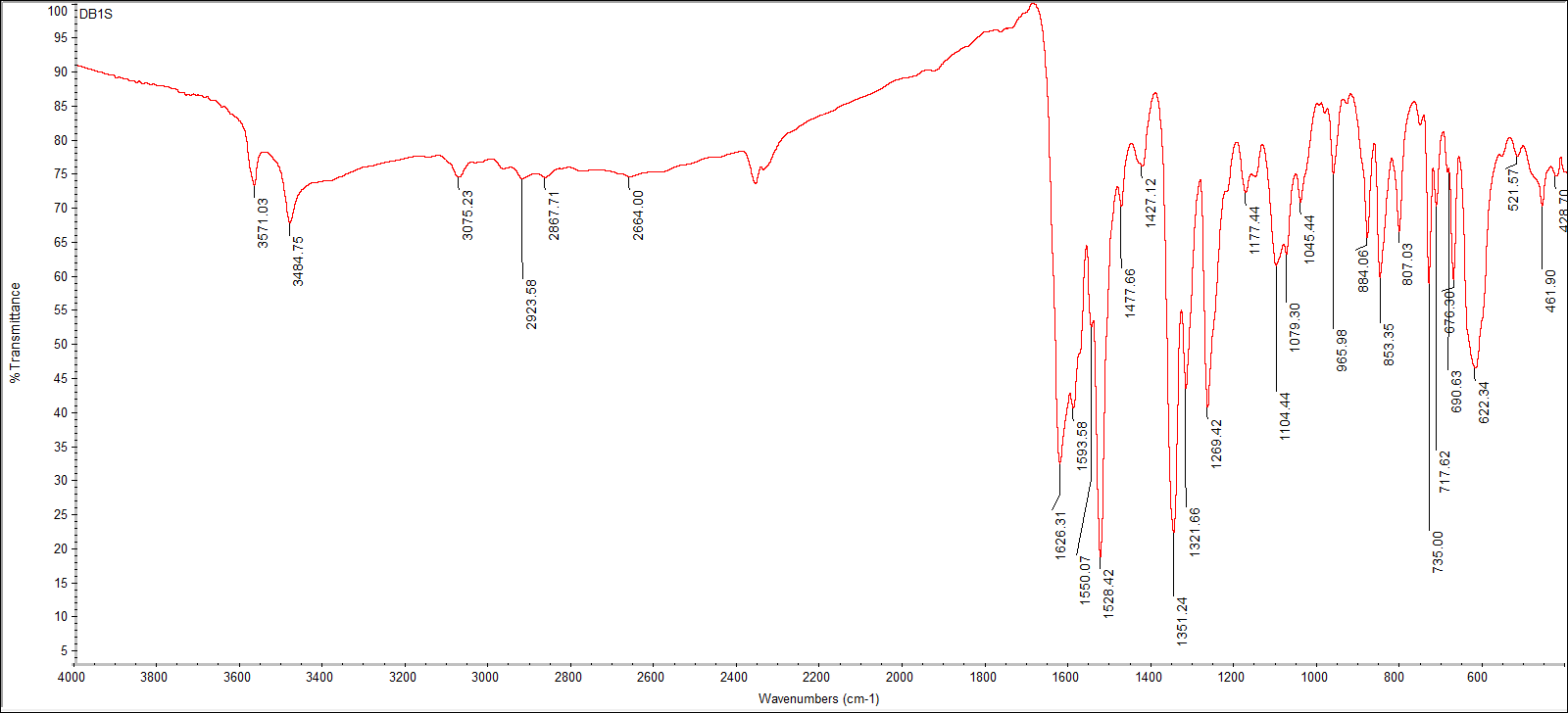** |

**Fig. S2. I**R spectra of the ligand and its complexes.

| **NBHD** |  |
| --- | --- |
| **1** |  |
| **2** |  |
| **3** |  |

**Fig. S3. UV-vis.** spectra of the ligand and its complexes.

|  |
| --- |
| **Fig. S.4.** Correlation plot between the EHOMO–ELUMO and the IC_50_ values for NBHD and its Cu(II) complexes (**1–3**) against HepG-2 cancer cells. |

| 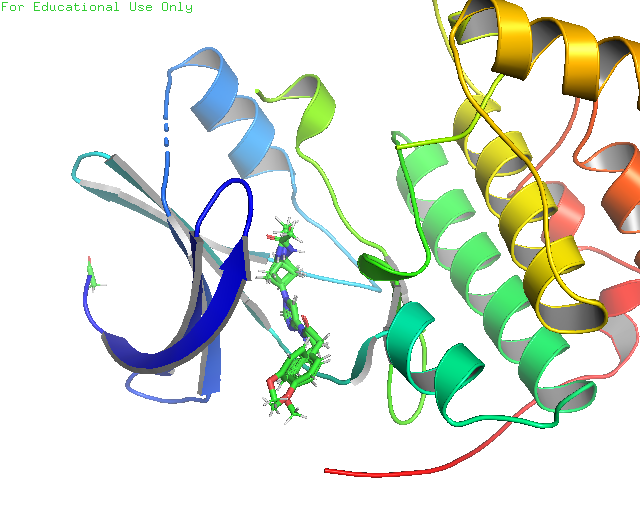 |
| --- |
| **Fig. S5.** 3D representation of the superimposition of the co-crystallized and the docking pose of the ligand in CDK-2 binding site. |

| **NBHD** | **1** |
| --- | --- |
| **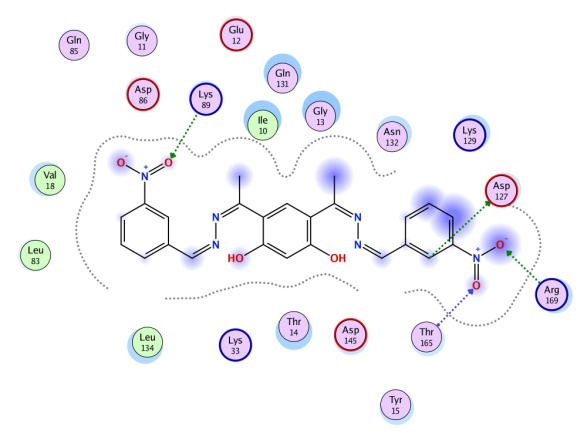** | 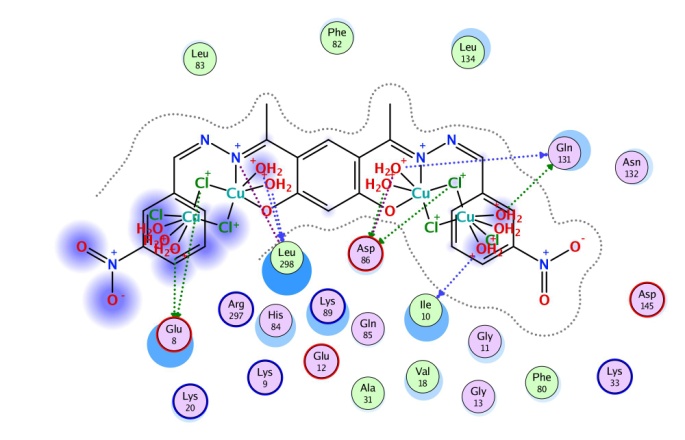 |
| **2** | **3** |
| **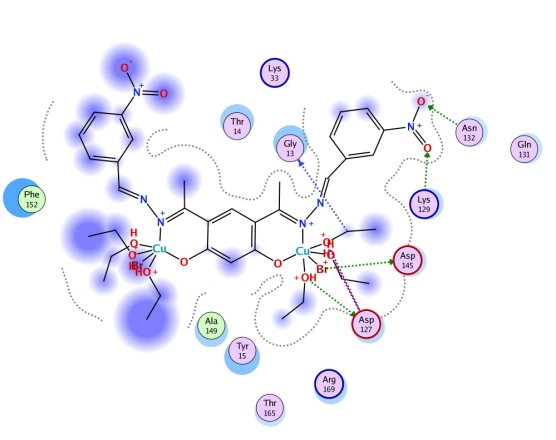** | 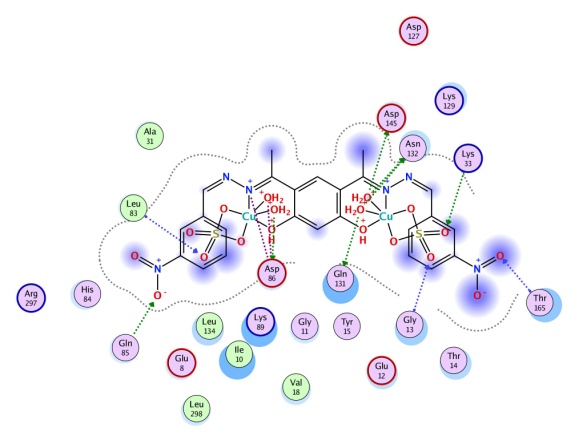 |
| **Fig. S6.** 2D interactions of docked compounds through the 3IG7. | |
